# Radiologic Evaluation of Bone Loss at Implants with Biocide Coated Titanium Abutments: A Study in the Dog

**DOI:** 10.1371/journal.pone.0052861

**Published:** 2012-12-21

**Authors:** Roberto López-Píriz, Eva Solá-Linares, Juan J. Granizo, Idohia Díaz-Güemes, Silvia Enciso, José F. Bartolomé, Belén Cabal, Leticia Esteban-Tejeda, Ramón Torrecillas, José S. Moya

**Affiliations:** 1 Nanomaterials and Nanotechnology Research Center-Consejo Superior de Investigaciones Científicas, Universidad de Oviedo, Principado de Asturias, Parque Tecnológico de Asturias, Llanera, Spain; 2 Advanced Oral Surgery Institute, Madrid, Spain; 3 Minimally Invasive Surgery Centre Jesús Usón, Cáceres, Spain; 4 Instituto de Ciencia de Materiales de Madrid-Consejo Superior de Investigaciones Científicas, Cantoblanco, Madrid, Spain; University of Toronto, Canada

## Abstract

The objective of the present study is to evaluate bone loss at implant abutments coated with a soda-lime glass containing silver nanoparticles subjected to experimental peri-implantitis. Five beagle dogs were used in the experiments, 3 implants were installed in each quadrant of the mandibles. Glass/n-Ag coted abutments were connected to implant platform. Cotton floss ligatures were placed in a submarginal position around the abutment necks and the animals were subject to a diet which allowed plaque accumulation, and after 15 weeks the dogs were sacrificed. Radiographs of all implant sites were obtained at the beginning and at the end of the experimentally induced peri-implantitis. The radiographic examination indicated that significant amounts of additional bone loss occurred in implants without biocide coating, considering both absolute and relative values of bone loss. Percentages of additional bone loss observed in implants dressed with a biocide coated abutment were about 3 times lower (p<0.006 distal aspect; and p<0.031 at mesial aspect) than the control ones. Within the limits of the present study it seems promising the use of soda-lime glass/nAg coatings on abutments to prevent peri-implant diseases.

## Introduction

Peri-implantitis is a common biological complication in implant therapy and a main cause of implant failure [1]–[4]. The presence of a submarginal biofilm induces a peri-implant inflammatory reaction which results in a breakdown of soft and mineralized tissues surrounding endosseous implants [5]. Findings from experiments in the dog and the monkey have demonstrated that submarginal plaque formation induced by ligature placement results in peri-implant tissue breakdown [6], [7].

Progression of peri-implantitis, when plaque formation is left untreated, is more pronounced in implants with rough surfaces than at implants with smooth surfaces [8] given that plaque formation is easier on rough surfaces. It is important to note the bacterial role in the peri-implantitis initiation and progression.

Concerning peri-implantitis some strategies have been developed in recent years [9]: On the one hand, efforts have been made in prevention of bone loss around implants-new implant designs have been commercialized seeking to reduce bone remodeling after osseointegration as well as modern implant-abutment connection (eg. morse cone-connection) minimizing bacterial filtration- although due to the impossibility of completely eliminating bacterial contamination, subgingival plaque formation is still a problem which often result in peri-implantitis; on the other hand, treatment remains based on mechanical debridation, antibiotic treatment and osseous regeneration when possible. However, it seems that the eradication of resistance is impossible and development of resistance to any particular antibiotic is inevitable.

A new approach to biomedical device-associated infections is based on biocide materials [10]. Silver as a nonspecific biocide agent is able to act strongly against a broad spectrum of bacterial and fungal species, including antibiotic-resistant strains. It is believed that silver nanoparticles (Ag NPs) are more reactive than bulk metallic forms because of the more active sites that result from a high specific surface [11], [12]. In this study, we have tested a soda-lime-glass containing Ag NPs-coated titanium abutments in an experimental peri-implantitis model, and its potential applications in reducing bone loss around implants.

## Materials and Methods

### Soda-lime-glass containing Ag NPs-coated titanium abutments

We have used a Soda-Lime-Glass/nAg powder to perform the coating on Ti-6Al-4 V alloy. The preparation of the starting powder and the characterization of the coatings were carried out according to the method developed by Esteban-Tejeda et al [11]. Homogeneous dispersed silver nanoparticles embedded into glassy matrix, with a content of silver of 20 wt.%, have been obtained as described below: A commercial soda-lime glass with the following chemical composition (mol.%): 70.30 SiO_2_, 0.92 B_2_O_3_, 15.34 Na_2_O, 7.62 CaO, 0.03 K_2_O, 4.78 MgO, 1.01 Al_2_O_3_, 0.01 Fe_2_O_3_, and the corresponding fraction of vitellinate-nAg [i.e., commercial protein with silver nanoparticles (batch n° 127, ARGENOL S.L.)] were homogeneously blended in isopropyl alcohol overnight under constant stirring. After the suspensions were dried at 60°C for 4 h, the homogeneous mixtures were uniaxially pressed into pellets (Ø∼10 mm) at 250 MPa. Next, they were sintered in two steps by heating to 500°C and to 725°C (rate of 3 C/min and dwell of 1 h), in order to ensure a complete elimination of the organic compounds from the vitellinate. The obtained glass pellets were milled down to <32 μm in an agate planetary mortar. These obtained powders were characterized by XRD, UV-VIS spectroscopy, scanning electron microscopy (SEM) and transmission electron microscopy (TEM) [13].

The green coating were obtained by dipping the Ti6Al4V abutments (Phibo ProUnic model, Spain) into a pentanol (Fluka-1-pentanol, 98.0% purity) glass-nAg powder suspension with 70 wt. % solid content. Before dipping, the suspension was dispersed in an ultrasonic bath and with a magnetic stirrer. During the coating process, the abutments were vertically dipped into the suspension at a constant speed of 500 mm/min, immersed into the suspension for 3 seconds, and then withdrawn at the same speed. The resulting coatings were dried at room temperature (20°C) for 24 h. The green coated abutments were subsequently heated in an argon atmosphere at 980°C for 1 h. The surfaces of the coatings, as well as polished cross sections, were examined by SEM (SEM, Phenom G2). The 3D roughness measurements (Ra, average roughness) were carried out with special software (3D Roughness Reconstruction, Phenom Pro Suit).

### Animals

**Figure 1:**
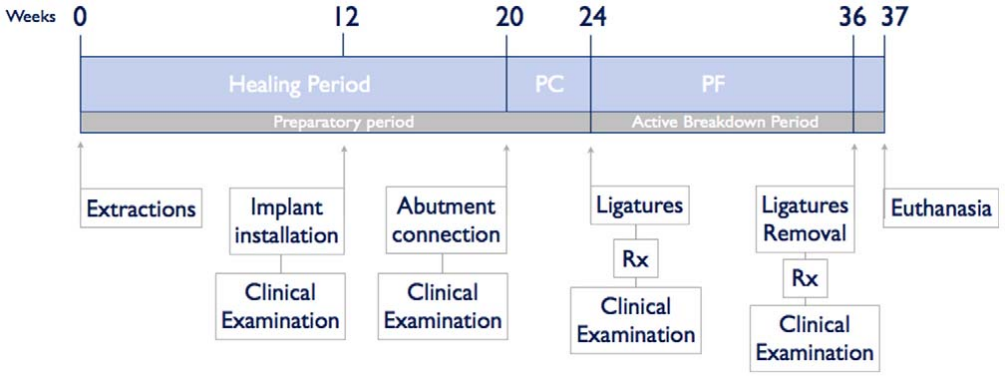
Outline of the study (**PC – Plaque control; PF – Plaque formation**)**.**

The study protocol was approved by the Ethics Committee for Animal Research Welfare, Minimally Invasive Surgery Centre, Cáceres, Spain. Five 1 year old Beagle dogs were used. The outline of the experiment is presented in Figure 1. During all procedures veterinary assistance was mandatory. General anesthesia was induced with intravenous injected propofol 10 mg/kg (Propofol Hospira, Hospira Productos Farmacéuticos y Hospitalarios, Madrid, Spain). A n°7 endotracheal tube with a balloon cuff was placed and connected to a circular anesthesia circuit (Leon Plus, Heinen & Löwenstein, Bad Ems, Germany). The anesthesia was sustained with sevofluorano (Sevorane, Abbott Laboratories, Madrid, Spain). Multimodal analgesia was employed in the perioperatory (ketorolac 1 mg/kg (Toradol 30 mg, Roche); – tramadol 1.7 mg/kg (Adolonta inyec., Grünenthal); y – buprenorfine 0,01 mg/kg (Buprex, Reckitt Benckiser Pharmaceuticals Limited, Berkshire, UK).

### Surgery

All mandibular premolars and the first molar were extracted. After three months of healing, mucoperiosteal flaps were elevated and 3 fixtures (Phibo Dental Solutions®, Barcelona, Spain; TSA Advance: length 11.5, diameter 3.75) were installed in the edentulous region on both sides of the mandible. A total of 30 implants were placed in the five dogs. During this period animals were feed with a soft diet. Two months later, abutment connection (ProUnic® Advance, Phibo; height 2 mm) was performed. These intermediate 2 mm height machine titanium surface abutments where placed to respect biological width, and over them healing abutments were placed. The mesial implants of each quadrant supported a machined titanium healing abutment were considered. The central and distal implants in each quadrant dressed biocide coated titanium healing abutments, and were considered case implants.

A plaque control program was initiated. This included cleaning of teeth and implants, once a day, 5 days a week, with toothbrush and dentifrice. The plaque control regimen was terminated four weeks later. At the end of the plaque control period, the animals were exposed to a clinical and radiographical examination. When no direct contact was observed between the marginal peri-implant soft tissues and the coated healing abutment, the height of the ProUnic® Advance abutment was reduced to 1 or 0 mm in order to assure a submarginal position of the biocide coated. Digital radiographs were obtained from all implant sections at the beginning and the end of experimental induced peri-implantitis. We used a holder that allowed easy and predictable alignment of the X-ray tube, and reproducible radiographic images from which highly repeatable measurements could be made. The radiographs were analyzed using Nemotec Dental Studio® Software (Nemotec SL, Madrid, Spain) and the distance between the abutment-fixture junction (A/F) and the marginal position of bone-to-implant contact (B) was determined. The measurements were made at both the mesial and the distal aspect of each implant and were redone after 1 week to confirm intra-observer reliability. The intra-examiner variability of radiographic measurements (mm) revealed small differences between the two assessments (mean 0.12). The variance and standard deviation were 0.08 and 0.2 respectively.

### Experimental peri-implantitis

Cotton ligatures were placed in a submarginal position around the neck of the fixture abutments according to the technique described by Ericsson et al [14] and Lindhe et al [6]. The plaque control regimen was finished and thus the plaque was allowed to accumulate during the course of the following three months. Once a week a clinical examination was performed to assess the plaque, soft tissue inflammation and presence of ligature. The ligatures were substituted every three weeks with new ligatures placed in the pocket of the receded gingival margin. At the end of the experimental peri-implantitis a new radiographic and clinical examination was performed. Animals were euthanized with a lethal dose of Sodium-Penthotal®, mandibular blocks containing fixtures were retrieved and stored in a 5% formaldehyde solution (pH 7). The implants were individual retrieved from the jaw bone using an oscillating autopsy saw. The retrieved specimens were immediately immersed in a solution of 4% formaldehyde and 1% calcium. The specimens were embedded in methyl-methacrylate and stained with combined basic fuchsin and toluidine blue. Transverse sections perpendicular to the beagle bone jaw with a thickness of approximately 15 μm were obtained for descriptive histology. The preparations were examined by using a transmitted light microscope (Optiphot, Nikon, Japan) equipped with a digital Camera (DP-12, Olympus, Japan).

### Statistical analysis

Mean values for all variables were calculated for mesial and distal aspect of each implant. Comparisons were made using absolute values (initial and final bone loss) and changes in a relative scale ((initial-final)/initial) (Table 1). Differences were analyzed using non-parametric (Mann-Whitney; Wilcoxon) methods. The null hypothesis was rejected at p≤0.05.

**Table 1:** Mean values for all variables for mesial and distal aspect of each implant.

	Implant*	N	Mean	SD	Mann-Whitney**
Distal Aspect Star Point	1	19	1.0374	0.38824	0.062
	0	10	0.7810	0.34301	
Mesial Aspect Star Point	1	19	1.08668	0.41696	0.085
	0	10	0.8150	0.37456	
Change Variable Distal Aspect	1	19	−0.90158	0.509327	<0.001
	0	10	−1.92400	0.777435	
Change Variable Mesial Aspect	1	19	−1.13000	0.563462	0.045
	0	10	−1.82300	0.894738	
Reduced Variable Distal Aspect	1	19	−1.32531	1.558999	0.006
	0	10	−3.47447	3.077830	
Reduced Variable Mesial Aspect	1	19	−2.04426	3.723847	0.031
	0	10	−3.73327	4.545198	

* Implant: 1 =  case implant; 0 =  control implant.

** Similar variances are assumed.

Change Variable (Distal or Mesial) =  Initial mean value – Final mean value.

Reduced Variable (Distal or Mesial)  =  (Initial mean value – Final mean value)/Initial mean value.

## Results

**Figure 2:**
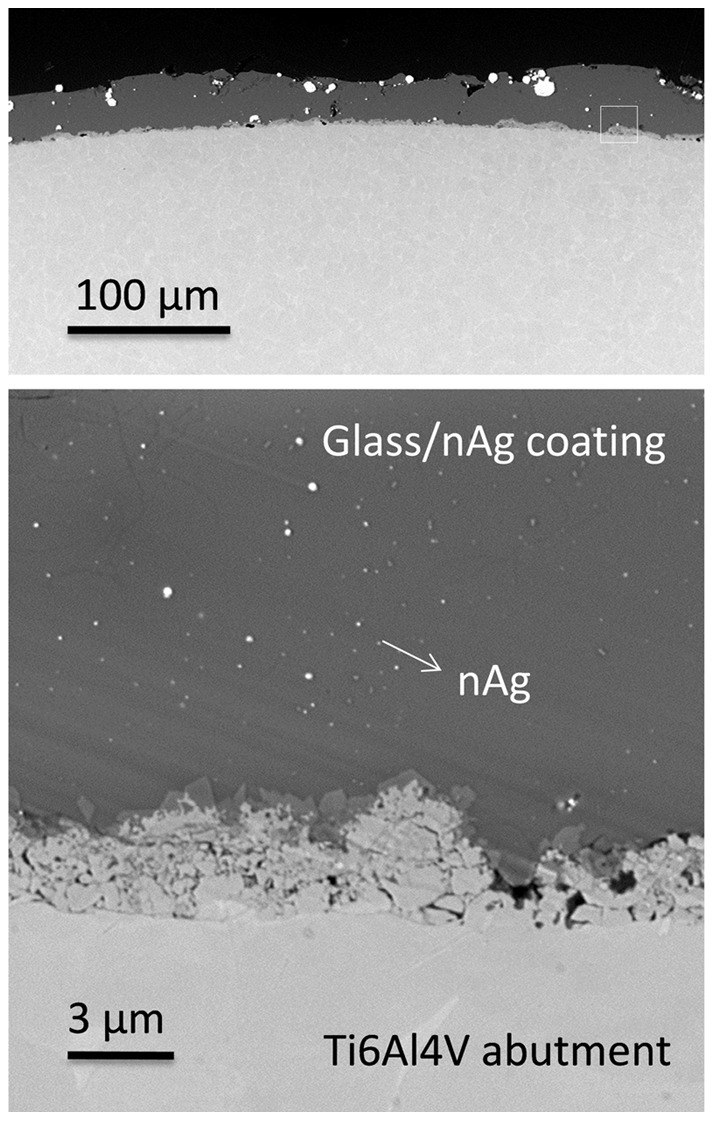
Scanning electron micrographs at different magnifications of cross section of the abutment.

A SEM image of a polished cross section of the abutment is shown in Figure 2. During firing at 980°C the soda-lime glass containing silver nanoparticles has flown wetting the metal surface and establishing a strong joining with the abutment surface [11]. The silver particle size ranges between 20–90 nm but also some agglomerates (0.5–8 µm) are present. The coating thickness was found to be ≈30 µm. Some defects and cracks can be observed.

**Figure 3:**
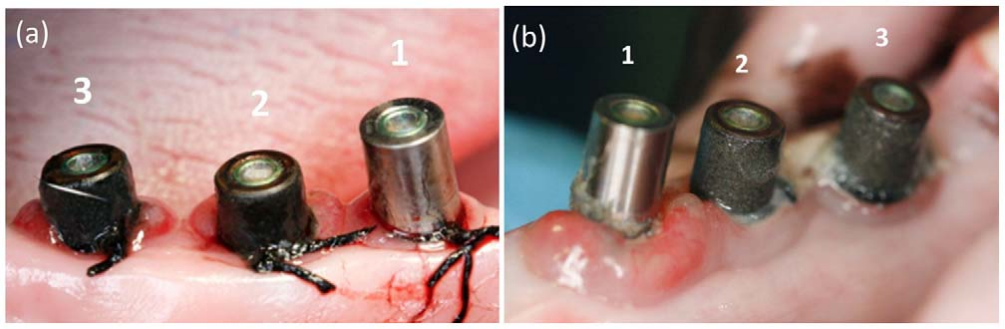
Images for the control implant (**1**) **and for the coated implants** (**2 and 3**)**.** (**a**) **week 25 and** (**b**) **week 36.**

At the second stage surgery one implant planned to be used as a case-implant was lost (dog 794 microchip code). Therefore a total of 29 implants were successfully osseointegrated. The clinical examinations performed at the initial weeks of the experimental induced peri-implantitis revealed important gingival inflammation around the biocide coated abutments and no changes in the control implants. These observations were progressively changing over, so that at the end of the experiment the control implant presented important gingival inflammation while biocide coated implants showed minimal gingival changes (Fig. 3). A large amount of plaque was harbored at the control implants while at the biocide coated implants plaque retention was located at ligature, despite the rough surface of the coating.

The mean amount of bone loss that occurred during the preparatory period (implant insertion-ligature placement) was 1.03 mm±0.388 at distal aspect and 1.08±0.416 at mesial aspect of case implant; and 0.781±0.343 at distal aspect and 0.815±0.374 at mesial aspect of control implant (Table 1). There are no significant differences in bone loss between case and control implants at this initial period (p = 0.062 in distal aspect and p = 0.085 in mesial aspect, Mann-Whitney).

**Figure 4:**
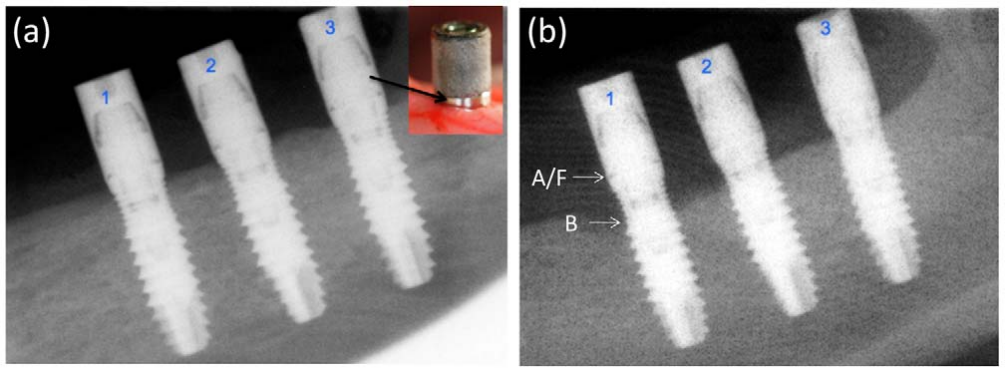
Digital micrographs for the control implants. (1) and coated implants (2 and 3). (a) week 24 and (b) week 36. A picture showing the intermediate 2 mm height machine titanium surface abutment has been inserted. The distance between the abutment-fixture junction (A/F) and the marginal position of bone-to-implant contact (B) is shown.

During the active breakdown period (experimental induced peri-implantitis) additional bone loss occurred (Fig. 4). This additional bone loss varied considerably between control and case implants (Table 1). In an absolute scale, the change variable (initial mean value – final mean value) shows a significant additional bone loss at control implants in distal aspect (p<0.001) and mesial aspect (p = 0.45). When we used a relative scale ((initial-final)/initial) to quantify additional bone loss during active breakdown period a percentage change of 347% was observed in distal aspect of control implants and 373% in mesial aspect. Percentages of additional bone loss observed in implants dressed with a biocide coated abutment were about 3 times lower (p<0.006 distal aspect; and p<0.031 at mesial aspect) than the control ones, as can be deduced from Table 1.

**Figure 5:**
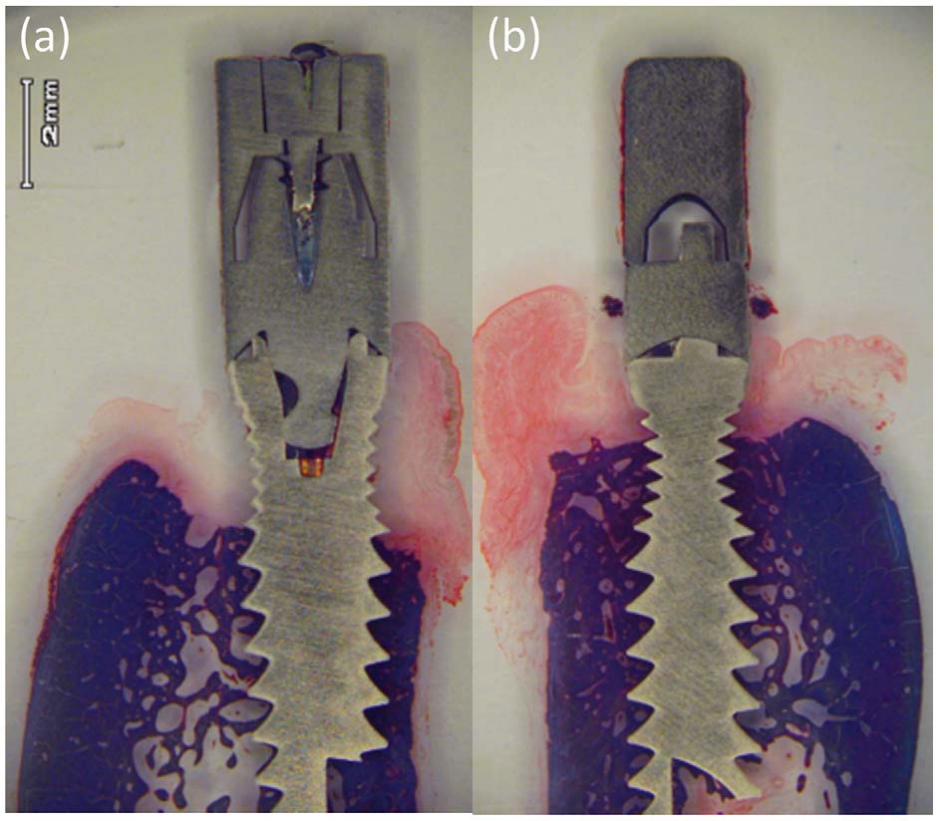
Transverse section of implants at the end of experiment. (a) Implant without coating and (b) implant coated by glass/nAg.

The fraction of bone loss observed in the corresponding implants transverse sections (Fig. 5) was found to be very similar to the one determined by digital radiographs (Fig. 4). A completed histological and histomorphometric analysis of the different transverse sections obtained from the implants is in progress.

## Discussion

In the present study bone loss that occurred at implants subjected to experimental peri-implantitis was analyzed. The different types of abutment included in the experiment, which differed with regards to presence or absence of glass/nAg biocide coating, were related with the exhibited loss of supporting bone at implants. Progression of peri-implantitis was more pronounced at implants wearing smooth machined titanium abutment (control implants) than at implants with glass/nAg biocide coated abutment (case implants).

The classical experimental model of peri-implantitis described by Ericsson et al [14] was applied in the present experiment. Thus, the ligatures were initially forced into a position apical to the mucosal margin and a “pocket” between the implant and the mucosa was thereby created. As previously demonstrated [6], the mechanical trauma produced by the ligatures together with plaque accumulation resulted in the establishment of large inflammatory lesions in the adjacent peri-implant tissues and substantial bone loss, but with significant differences between control and case implants.

In the present study, control implants developed large inflammatory lesions in the adjacent soft tissues, in contrast with case implants that only showed moderate inflammatory changes at the beginning of experimentally induced peri-implantitis (Fig. 3). Inflammatory changes in soft tissues around case implants took place in coincidence with a supplementary event: the removal of the 2 mm height abutments and direct connection between coated healing abutment and implant platform. This constituted an insult to biological width, but was considered to be necessary in order to ensure a direct contact between biocide coating and peri-implant soft tissues. A few weeks after this procedure, inflammatory lesions around case implants remitted whereas in control implants these increased weekly. The reason for soft tissue inflammation around case implants in the initial weeks of the active breakdown period was probably related with the insult to the biological width and the consequent peri-implant tissue remodelation more than with the ligature induced peri-implantitis, since the ligatures did not cause significant inflammation around control implants at this stage of the procedure. It follows from this reasoning that the rapid resolution of the initial inflammatory reaction in case implants may indicate the establishment of a new biological width, especially when considering that active breakdown by ligatures caused a progressive increased inflammatory reaction only reveal in control implants. There is reason to believe that glass-nAg coated abutments may prevent soft tissue inflammatory reactions.

**Figure 6:**
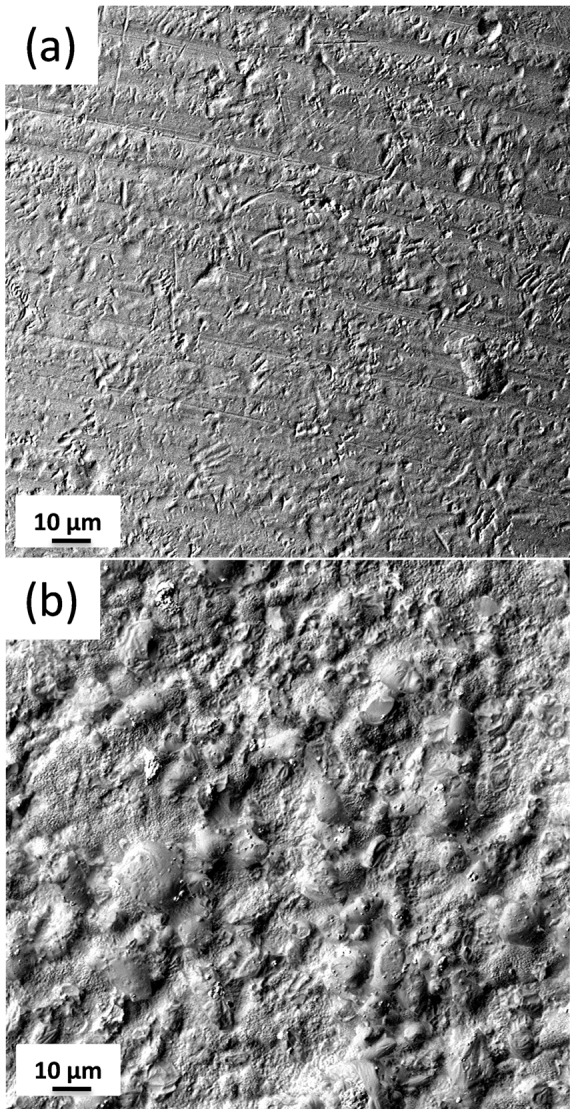
Scanning electron micrographs of the surface for (**a**) **titanium implant and** (**b**) **coated/nAg titanium implant.**

In the study by Berglundh et al [8] it was concluded that peri-implantitis was more pronounced at the implants with the rough surface than at the implants with the smooth surface. In the present study the same commercially available implants were used, but there was a large difference in surface roughness between control and biocide coated healing abutments. Representative SEM surface topographies of the samples are shown in Figure 6. About twice average Ra values were found for silver doped glass coated abutments (1±0.2 µm) versus titanium abutments (0.5±0.3 µm). What should have been considered an important disadvantage for case implants had not the expected findings according to previous experiments. This was due to the biocide effect of glass-nAg coating which avoids plaque adhesion to its surface as it has been demonstrated in previous experiments on coated Ti-6Al-4V plates, additionally the quantitative evaluation of silver release pointed out the low toxicity and long-term effectiveness of the coating. [15], [16].

In the present experiment bone remodeling around control and case implants at the beginning of the active breakdown peri-implantitis did not show significant differences (p<0.062 and p<0.085 at distal and mesial aspect respectively). But at the end of the induced peri-implantitis period significant differences were observed between control and case implants, not only in both mesial and distal aspect of implants, but also when considering absolute or relative variables (Table 1, Figure 4 and 5). Significantly large amounts of bone loss occurred at control implants when compared with implants connected to a glass/nAg coating healing abutment. The present study clearly documented that glass/nAg coated abutment decreases significantly bone loss due to peri-implant breakdown.

Differences in bone loss were more significant at distal aspect than at mesial aspect of all implants. The reason for this difference is not fully understood. The insult to biological width of case implants at week 24 deserves special consideration, because it may explain a new tissue remodeling process around case implants and subsequent periimplant bone loss not due to ligature induced peri-implantitis. Although this event may have had a negative influence in case implants results, statistical differences and significances between control and case implants are large enough to demonstrate that glass/nAg coating reduces bone loss in peri-implantitis. There is strong reason to believe that preservation of initial biological width in case implants had lead to no bone loss at all in implants with glass/nAg coating.

## Conclusions

In the present study, clinical examinations showed that implants with the soda-lime glass/nAg coating presented minimal inflammatory changes in soft tissues surrounding implants compared with control ones. Further, our analysis indicates that radiologic bone resorption around implants due to experimentally induced peri-implantitis is significantly large in control implants when compared with implants dressed with the glass/nAg silver coatings.

As it is evident from the results presented in this study, the glass/nAg coatings emerge as a promising approach to prevent peri-implantitis. There is a need for an analysis of the clinical course of bone resorption around coated implants placed in patients with high risk of peri-implantitis. Therefore, clinical trial designs dealing with glass/nAg coatings are an urgent must.
